# Conventions and workflows for using *Situs*

**DOI:** 10.1107/S0907444911049791

**Published:** 2012-03-16

**Authors:** Willy Wriggers

**Affiliations:** a Department of Physiology and Biophysics and Institute for Computational Biomedicine, Weill Medical College of Cornell University, 1300 York Avenue, New York, NY 10065, USA

**Keywords:** *Situs*, multi-resolution data

## Abstract

*Situs* is a modular program package for the multi-scale modeling of atomic resolution structures and low-resolution biophysical data from electron microscopy, tomography or small-angle X-ray scattering. This article provides an overview of recent developments in the *Situs* package, with an emphasis on workflows and conventions that are important for practical applications. The modular design of the programs facilitates scripting in the bash shell that allows specific programs to be combined in creative ways that go beyond the original intent of the developers. Several scripting-enabled functionalities, such as flexible transformations of data type, the use of symmetry constraints or the creation of two-dimensional projection images, are described. The processing of low-resolution biophysical maps in such workflows follows not only first principles but often relies on implicit conventions. *Situs* conventions related to map formats, resolution, correlation functions and feature detection are reviewed and summarized. The compatibility of the *Situs* workflow with CCP4 conventions and programs is discussed.

## Abstract 2

Recent developments of the *Situs* software suite for multi-scale modeling are reviewed. Typical workflows and conventions encountered during processing of biophysical data from electron microscopy, tomography or small-angle X-ray scattering are described.

## 1. Introduction

*Situs* is a modular command-line-based open-source package written in C/C++ and is available under the GNU GPL License. Originally designed in 1998/9 to assist in the visual­ization and interpretation of cryo-electron microscopy (cryo-EM) density maps (Wriggers *et al.*, 1999 ▶), its scope has been extended over the years to model multi-resolution data from a variety of biophysical sources, including tomography and small-angle X-ray scattering (SAXS; Wriggers & Chacón, 2001*b* ▶). The usefulness of hybrid multi-scale methodologies that combine atomic structures with lower resolution density maps or coarse-grained models has been well established (Mendelson & Morris, 1997 ▶; Lindert *et al.*, 2009 ▶) and the historical evolution of *Situs* with application examples was reviewed in Wriggers (2010 ▶).

The focus of this paper is on practical applications, specifically on workflows and conventions that are implicit to *Situs* programs. Over the years, owing to the modular design, the number of possible combinations of programs has increased to a point where it has become difficult to document possible workflows in our online tutorials. Based on specific modeling tasks, some new usage examples are provided here to inspire users to experiment on their own. Also, in an effort to bridge *Situs* to other software, many of the implicit conventions are described for the first time.

**Figure 1:**
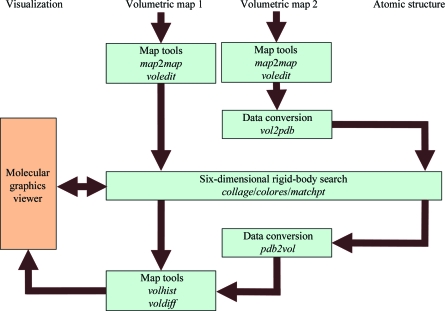
Modular design of the *Situs* package. Major *Situs* components (blue) are classified by their functionality. The main workflow is indicated by brown arrows. The visualization (orange) for the rendering of the models requires an external molecular-graphics viewer compatible with the density-map format, such as *VMD* (Humphrey *et al.*, 1996 ▶), *Chimera* (Pettersen *et al.*, 2004 ▶) or *Sculptor* (Birmanns *et al.*, 2011 ▶). This example workflow shows how various *Situs* tools can bring two volumetric maps into register (see text). Since *Situs* rigid-body matching tools require a PDB file for the docking to a target map, the second map is intermittently transferred into atomic (PDB) format. After the matching of the pseudo-atomic map, it is interpolated back into volumetric format and can then be processed further.

Our online tutorials (http://situs.biomachina.org) now include Unix bash-shell scripts for the automatic generation of tutorial solutions. Much of the workflow complexity originates from the command-line-based scripting that allows programs to be combined in creative ways. Fig. 1 ▶ shows a typical example. The hypothetical problem is that one would like to bring two volumetric density maps into register. The maps can be format converted (§[Sec sec2]2) with the *map*2*map* tool or processed with ‘volume algebra’ tools (§[Sec sec8]8) such as *voledit*. For technical reasons, the rigid-body matching tools *collage*, *colores* and *matchp(oin)t* require an atomic PDB file for docking to a target map (Fig. 1 ▶). Therefore, the second map must be intermittently transformed to the atomic (PDB) format using *vol*2*pdb* so it is free from the cubic lattice (for the rotation and translation in the docking). After the matching of the pseudo-PDB map, it can be interpolated back into the volumetric format through projection onto the original lattice with *pdb*2*vol*.

Many of the *Situs* tools rely on implicit conventions for setting parameters. It is perhaps surprising to readers from the crystallographic community that important parameters of volumetric density maps such as resolution, density levels and even map formats are not strictly defined in the hybrid modeling community. For example, the surface isolevel used for visualizing a volume map is an intuitive concept that is surprisingly difficult to solve computationally. Although one can attempt to set the isolevel based on the enclosed volume (Harpaz *et al.*, 1994 ▶), the resolution lowering leads to a shift in density, eroding convex features and filling up concave features of the atomic structure. To prevent convex features from protruding from the low-resolution surface, the isolevel of cryo-EM maps is empirically set to enclose 120–150% of the molecular volume depending on the overall shape of the system. This is just one example where empirical ‘fudge factors’ trump first principles. Over time, software developers have implemented conventions for a multitude of such quantities as they were breaking new ground. Although an effort is under way to standardize such conventions (Heymann *et al.*, 2005 ▶), it is still often necessary to investigate the source code when sharing data between different software packages. In an effort to create more transparency, the most important *Situs* conventions are documented here.

The remainder of this paper is organized as follows. §[Sec sec2]2 describes the evolution of the *Situs* and CCP4-based map formats. §[Sec sec3]3 exemplifies the conversion between low-resolution structure types using small-angle X-ray-related bead models. §[Sec sec4]4 contains a comparison of resolution conventions used for multi-scale biophysical data. §[Sec sec5]5 describes conventions for coarse-grained models used in structure matching. §[Sec sec6]6 presents correlation-based fitting approaches and the conventions used for computing the cross-correlation. §[Sec sec7]7 presents workflows enabled by shell scripting, such as the implementation of symmetry constraints and two-dimensional projection. The paper concludes with a discussion of extended and supplemental *Situs* functions in §[Sec sec8]8.

## 2. Map format conventions

In this section, we describe three-dimensional density-map formats directly supported by *Situs*, such as the original *Situs* map format and the conventions regarding various CCP4-derived formats developed at MRC Cambridge, England.

Volumetric maps (Fig. 1 ▶) come in a variety of map formats, many of which were inspired by crystallographic formats. For example, the *map*2*map* format-conversion utility of *Situs* accepts cryo-EM and crystallographic density files in ASCII (text), CCP4, MRC, *Situs, SPIDER* and *X-PLOR* formats, automatically adjusts to the machine architecture (endianism) and supports permutation of axes as well as non-orthogonal unit cells (this is accomplished by trilinear interpolation to a cubic lattice, if necessary). In the late 1990s there were no universally accepted standards in the cryo-EM community for setting the origin of the map coordinate system, which is critical for the docking of atomic structures. The minimalist *Situs* format was conceived to keep track of the coordinate system, to enforce a cubic lattice and to be independent of the ever-changing map-format standards in the community. Although this ASCII-based (and thus easily readable/editable) format was initially meant to be of limited use only within the *Situs* package, it is now supported by the molecular-graphics programs *VMD* (Humphrey *et al.*, 1996 ▶), *Chimera* (Pettersen *et al.*, 2004 ▶) and *Sculptor* (Birmanns *et al.*, 2011 ▶), by the *EMAN*2 reconstruction package (Ludtke *et al.*, 1999 ▶) and also by the *em*2*em* format-conversion tool (http://www.imagescience.de/em2em). In the map format, a short header holds the voxel spacing WIDTH, the map origin as defined by the three-dimensional coordinates of the first voxel ORIGX, ORIGY, ORIGZ and the map dimensions (number of increments) NX, NY, NZ. This minimalist header is followed by the data fields such that *x* increments change fastest and *z* increments change slowest.

To take advantage of a more compact binary data storage, CCP4 and MRC file formats were recently adopted for direct use within *Situs* programs, eliminating the *map*2*map* format-conversion step from the workflow for many users. The rendering of the map formats was coordinated with the developers of *em*2*em* (Michael Schatz), *Chimera* (Tom Goddard), *VMD* (John Stone) and *Sculptor* (Stefan Birmanns). We have made sure that the *Situs* format matches all other format conventions, such that different map formats and PDB structures are rendered consistently. This effort required, in particular, a revisiting of the CCP4-derived map formats developed at MRC Cambridge, which have changed and diversified over the years. The MRC file format was once identical to the CCP4 format; however, incremental changes and the lack of utility of some *CCP*4 features in the EM community have caused them to become incompatible. Details of the various CCP4 and MRC map formats, and the *Situs* conventions for reading them, are provided as Supplementary Material^1^. Our recent efforts were timely because the CCP4 format, as generated by *em*2*em*, has become the official format of the EMDB map data bank (Tagari *et al.*, 2002 ▶). The detailed conventions we arrived at are described in the Supplementary Material^1^ (pp. 7–8). We hope that our conventions will be more widely adopted and that they help to ensure that maps display correctly.

## 3. Structure-type conversion

*Situs* supports three multi-resolution structure types: atomic structures (PDB format), volumetric density maps (§[Sec sec2]2) and SAXS bead models (PDB format). Fig. 1 ▶ shows how to interconvert between atomic and volume data.

(i) *vol*2*pdb* allows one to encode positive density values of a three-dimensional map into a PDB file, with the densities written to the PDB occupancy field.
(ii) *pdb*2*vol* is a real-space convolution tool. It allows one to lower the resolution of an atomic structure to a user-specified value or to create a bead model from atomic coordinates. The structure is first projected onto a cubic lattice using trilinear interpolation. Subsequently, each lattice point is convoluted with one of several supported kernel (point-spread) functions, *e.g.* Gaussian, triangular or hard sphere (see the online user guide).

**Figure 2:**
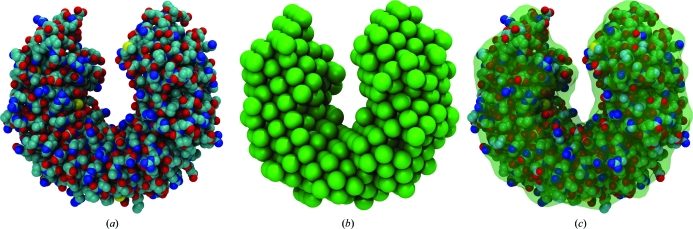
Real-space fitting and visualization of SAXS bead models with *Situs* (Wriggers & Chacón, 2001*b* ▶; Wriggers, 2010 ▶). (*a*) Atomic structure of ribonuclease inhibitor (PDB entry 1bnh; Kobe & Deisenhofer, 1996 ▶). (*b*) Coarse-grained bead model (393 beads, 3 Å radius, hexagonal close-packed) generated from (*a*) with the *Situs* tool *pdb*2*sax*. (*c*) Atomic structure fitted to the bead model using *collag*e. To show the embedded structure, the bead model is rendered as a transparent envelope. The envelope is the half-maximum isosurface of a density map created from the beads with *pdb*2*vol* using Gaussian kernel convolution with (half-maximum) kernel radius 3 Å. The images were rendered with Tachyon ray tracing using *VMD* (Humphrey *et al.*, 1996 ▶). For an updated review of the complete SAXS workflow, see Wriggers (2010 ▶) and the SAXS tutorial at http://situs.biomachina.org.

*Situs* also supports three-dimensional bead models from SAXS (Chacón *et al.*, 2000 ▶). Fig. 2 ▶ shows an application of SAXS-related tools in the visualization and atomic interpretation of SAXS-derived shapes (Wriggers & Chacón, 2001*b* ▶). To test the docking accuracy, the *pdb*2*saxs* tool was created, which projects atomic structures onto a hexagonal close-packed lattice, with user-defined bead radii written to the PDB occupancy field. The resulting models (Fig. 2 ▶ *b*) served as ‘simulated’ low-resolution data in Wriggers & Chacón (2001*b* ▶).

The bead models in PDB format can be transformed into density maps for subsequent docking using a hard-sphere kernel in *pdb*2*vol*. The SAXS modeler then has access to all docking strategies supported by *Situs*, including correlation-based docking (*collage* or *colores*), feature-point based matching (*matchpt*) and even flexible real-space fitting (§[Sec sec8]8).

One problem in the interpretation of SAXS data is the visualization of the beads. It is useful to render not the densely packed beads themselves (Fig. 2 ▶ *b*), but a transparent wire mesh or envelope (Fig. 2 ▶ *c*) that shows the fitted atomic structure. This envelope was created by isocontouring an intermediate volumetric map, which was generated from the beads by convolution with a soft kernel such as a Gaussian (using *pdb*2*vol*).

Our approach to the rendering and interpretation of SAXS data has been adopted by other groups in the structural biology community (Lipfert *et al.*, 2007 ▶; Fagan *et al.*, 2009 ▶; Alvarado *et al.*, 2009 ▶). More details, including a workflow and a listing of programs that are used to dock an atomic structure into low-resolution SAXS models, are provided in an online tutorial (http://situs.biomachina.org/tutorial_saxs.html).

## 4. Resolution convention

This section relates the resolution convention used in *Situs* with the crystallographic resolution and the well known Rayleigh point resolution in optics.

Resolution *R* is a quantity in Fourier space and has dimension Å^−1^, but the real-space quantity *r* = *R* ^−1^ is also often termed ‘resolution’ in biophysical parlance. The symbols *r* and *R* are used here to differentiate between the spatial and frequency domain.

In crystallography, let *R*_c_ be the radius of a circular region within which Fourier terms contribute to the crystallographic synthesis; one can then say that *r*_c_ = *R*_c_^−1^ is the crystallo­graphic resolution (Frank, 2006 ▶). In contrast, Rayleigh con­sidered the resolvability of two points in real space (Stenkamp & Jensen, 1984 ▶). The real-space image of one point, whose Fourier transform is limited by a disk of radius *R*_c_, is the Airy pattern [*J*_1_(2π*rR*_c_)/(2π*rR*_c_)]^2^, where *J*_1_ is the first-order Bessel function. The Rayleigh criterion is satisfied when the first minimum of one point’s Airy pattern coincides with the central maximum of the other. This critical point-to-point distance *r*_p_ turns out to be *r*_p_ = 0.610*r*_c_ (see Appendix *A*3 in Radermacher, 1988 ▶). The Airy pattern is dominated by a bell-shaped central Airy disk, which measures a ‘full-width at half-maximum’ FWHM = 0.514*r*_c_. Ignoring the weak outer rings (resulting from the hard limit in Fourier space), the Airy pattern can be approximated by a Gaussian function that matches the Airy disk (http://en.wikipedia.org/wiki/Airy_disk).

In the development of the *pdb*2*vol* tool of *Situs* in the 1990s, the width of the Gaussian convolution kernel was set empirically to mimic cryo-EM maps at published resolution values (for resolution estimation in cryo-EM, see Frank, 2006 ▶). The empirical *Situs* resolution *r*_s_ was set to *r*_s_ = 2σ (or 1.471 FWHM), where σ is the three-dimensional standard deviation of the Gaussian kernel, exp(−3*r* ^2^/2σ^2^). One can relate the *Situs* resolution *r*_s_ to the crystallographic resolution *r*_c_ and to the point resolution *r*_p_ by matching the Gaussian to the Airy disk of the same FWHM. It follows that *r*_s_ = 1.239*r*_p_ = 0.756*r*_c_. Note that the *Sculptor* molecular-graphics software (Birmanns *et al.*, 2011 ▶) from our laboratory also follows this convention.

The *pdb*2*mrc* tool of the *EMAN* package (Ludtke *et al.*, 1999 ▶; renamed *e2pdb2mrc.py* in *EMAN*2) is another popular resolution-lowering tool which established its own resolution measure, *r*_e_. In *EMAN*, the functional form of the Gaussian real-space kernel is exp(−π^2^ *r* ^2^/*r*_e_^2^). The Fourier transform exp(−*r*_e_^2^/*R* ^2^) is not strictly limited to a hard radius, as in the crystallographic case, but a reasonable radius of the soft Gaussian in Fourier space is set by *EMAN* at the 1/*e* cutoff where *R* = *R*_e_. After matching the Gaussian real-space kernel to the Airy disk, it follows that *r*_e_ = 1.886 FWHM = 1.589*r*_p_ = 1.282*r*_s_ = 0.969*r*_c_.

In summary, both the *EMAN* and *Situs* resolution values are larger than the point resolution and smaller than the crystallographic resolution: *r*_p_ < *r*_s_ < *r*_e_ < *r*_c_. However, the *Situs* resolution *r*_s_ is closer to the Rayleigh criterion *r*_p_, whereas the *EMAN* resolution *r*_e_ is almost identical to the crystallographic resolution *r*_c_ as defined by Frank (2006 ▶). When comparing similar maps between *Situs* (or *Sculptor*) and *EMAN*, the resolution values *r*_s_ (in units of Å) are smaller by a factor of 1.282 compared with *r*_e_.

## 5. Next-generation feature-point-based matching

This section describes a recent paradigm shift in setting the level of detail of coarse-grained models to better represent resolved features of the structural data. Prior to *Situs* v.2.5, the level of detail of the coarse-grained model was limited by the algorithm, whereas now the level of detail is matched to the spatial resolution of the structures.

The idea of using coarse-grained models of feature points for the docking of multi-scale structures is a classic idea that predates *Situs* (Wriggers *et al.*, 1998 ▶). We have shown in earlier work that this approach is advantageous at resolutions below 10 Å because the points provide ‘interior features’ (and an encoding of the molecular shape) even in the absence of interior density variations from the secondary structure. However, the earlier *Situs* tools had a limited number of feature points and required both point clouds to be of equal size. We have recently published a new anchor-point registration technique that overcomes these combinatorial limitations and is able to dock smaller point clouds to larger ones (Birmanns & Wriggers, 2007 ▶).

**Figure 3:**
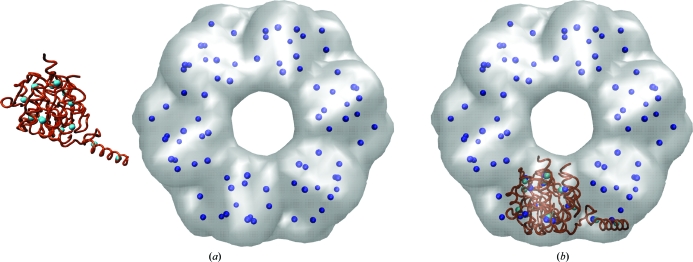
Rigid-body fitting of a RecA monomer (PDB entry 2rec, brown; Yu & Egelman, 1997 ▶) to a simulated 15 Å resolution map of the hexamer (gray) using point-cloud matching. (*a*) Before and (*b*) after matching with the *Situs matchpt* tool. The coarse-grained anchor points (feature vectors, cyan/blue) were generated using vector quantization (Wriggers *et al.*, 1998 ▶) in *matchpt*. The low-resolution map was generated with *pdb*2*vol* using a Gaussian kernel convolution. The images were rendered with *VMD* (Humphrey *et al.*, 1996 ▶).

Fig. 3 ▶ shows a typical application of feature-point-based rigid-body matching. The *matchpt* utility is a command-line program for matching arbitrary-sized three-dimensional point sets (coarse-grained models), which can be generated on the fly or by using the output of the *Situs* programs *qpdb* and *qvol*. The utility can dock a subunit into a larger target map, *i.e.* find *N* feature points within another set with *M* points, *N* < *M*, and match them. To solve this problem, *matchpt* uses a heuristic and investigates only a subset of all possible permutations of feature points (Birmanns & Wriggers, 2007 ▶).

The idea of matching point sets was based on the observation that for many low-resolution maps numeric values of the cross-correlation (CC) are often in a narrow range and less discriminatory compared with the r.m.s.d. values of the feature points (Wriggers *et al.*, 1999 ▶). This is a consequence of the fact that feature points can reliably and reproducibly encode the molecular shape, even in the absence of interior (secondary-structure) density features. Therefore, it makes sense for difficult low-resolution maps to use *matchpt* as an alternative to the CC-based tools *colores* and *collage*. In the default mode, a user would explore the quality of the match of the point clouds by minimizing their r.m.s.d. Alternatively, the minimum of the statistical variability (here the sum of average variabilities of both point sets) can be used to select an optimum *N* and *M*, since this variability was found to be a good estimator for the docking accuracy (Wriggers & Birmanns, 2001 ▶). Finally, a user may wish to explore the standard cross-correlation (CC), which is discretely sampled by the solutions of the point-cloud matching.

In *Situs* v.2.6, the *matchpt* tool was improved to replace all the functionality of the classic *Situs* tools *qdock* and *qrange*, and the online tutorials were updated accordingly. This introduced a paradigm shift for the way the level of detail of the coarse-grained models (*N* and *M*) is estimated. In situations where a smaller structure is docked into a larger density (*e.g.* an oligomeric map), the -units parameter defines the fraction of occupied volume (which may be non-integer), *i.e.* it estimates how many atomic input structures fit into the target volume. *M* is then defined as -units × *N*. To estimate the number *N* of feature points, one can divide the volume of the atomic structure by the volume of a resolution element, *r*_s_^3^ (where *r*_s_ is the resolution value of the target map in Å). This calculation gives an upper bound for the number of features *N* contained in the structure (and, *via M*, the number of features contained in the volume) at the given map resolution. To avoid overfitting and to find an optimal number, it is useful in practical applications to bracket *N* between the 30 to 50% level of this upper bound.

## 6. Next-generation correlation-based matching

This section highlights recent advances in correlation-based fitting technology and the underlying convention in the calculation of the CC.

The *Situs* cross-correlation coefficient (CC) for volumetric maps ρ_vol_(**r**) and ρ_calc_(**r**) is defined as
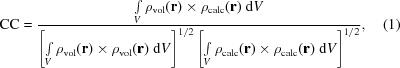
where ρ_vol_(**r**) is a low-resolution map and ρ_calc_(**r**) is an atomic structure subject to rigid-body movements, projection to the volume *V* and convolution with a Gaussian kernel (Wriggers & Chacón, 2001*a* ▶). The CC values are normalized, but unlike in the Pearson correlation coefficient the averages are not subtracted from the densities. Consequently, the calculation is slightly more efficient than that of the Pearson CC. The convention takes into account that ρ_calc_(**r**) often has the physical meaning of a density with well defined positive amplitude that corresponds to the represented low-resolution structure. This way, CC ∈ [0, 1] for ρ_vol_(**r**), ρ_calc_(**r**) > 0. One can show that maximizing the *Situs* CC value also maximizes the Pearson correlation coefficient, so dropping the subtraction of averages in (1) yields no performance penalty. The *Situs* CC convention has also been adopted by the *Sculptor* visualization program (Birmanns *et al.*, 2011 ▶).

Chacón & Wriggers (2002 ▶) introduced *colores*, a widely used CC-based fitting tool that takes advantage of Fourier correlation theory to rapidly scan the translational degrees of freedom of a probe molecule relative to a (fixed) target-density map (whereas the rotations are sampled exhaustively by the enumeration of a list of homogeneously distributed Euler angles). The performance of the standard CC (1) is limited to resolutions higher than 10 Å, where densities exhibit the internal (*i.e.* secondary) structure. The major advantage of *colores* is that it extends the viable resolution range to ∼30 Å by means of an (optional) Laplacian operator [applied to both ρ_vol_(**r**) and ρ_calc_(**r**) in equation 1] that emphasizes shape-contour information in addition to the traditional volume correlation (also, a masking filter was implemented that suppresses singularities of the Laplacian of ρ_vol_ at density edges resulting from thresholding or segmentation).

Recently, the new refinement tool *collage* was introduced which performs a conjugate-gradient optimization of the same scoring functions known from *colores*. The main innovation is the simultaneous optimization of multiple rigid fragments that ‘see’ each other and avoid steric clashes by means of the normalization in (1). Birmanns *et al.* (2011 ▶) showed that this approach yields more accurate fits. Also, if all density is accounted for by the fragments it is no longer necessary to use the Laplacian filter option, even at low resolution.

**Figure 4:**
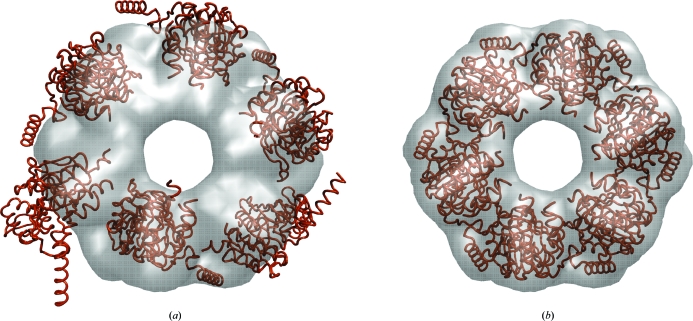
Multi-fragment refinement using *collage*. (*a*) RecA monomers (PDB entry 2rec, brown) in random start positions relative to the simulated 15 Å resolution map of the hexamer (see Fig. 3 ▶). (*b*) Final fit of the monomers. The images were rendered with *VMD* (Humphrey *et al.*, 1996 ▶). For the complete fitting workflow, see the multi-fragment docking tutorial at http://situs.biomachina.org.

Fig. 4 ▶ shows an application example of *collage* using the simultaneous optimization of six monomers. A single run of off-lattice Powell optimization is applied that refines a preliminary multi-fragment model (consisting here of six input PDB files) to the nearest maximum of the CC. The start model of fragments could, for example, be derived manually by eye in a graphics program (as was performed here) or it could be based on *colores* or *matchpt* solutions. We have created a new multi-fragment online tutorial to explain this powerful new approach.

## 7. Scripting-based workflows

This section describes the benefits of bash-shell scripting to tie together multiple tasks. Example scripts are provided here for the implementation of symmetry constraints in multi-fragment fitting and for the creation of two-dimensional projections from three-dimensional maps.

**Figure 5:**
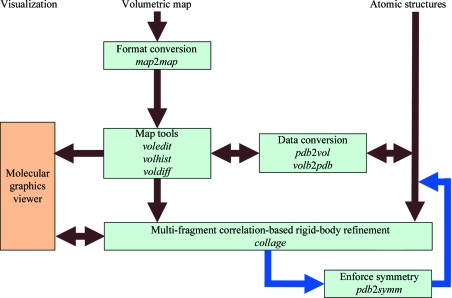
Schematic diagram of multiple fragment-related and symmetry-related routines in *Situs* (see text). The multi-fragment refinement tool *collage* requires one volume (target) and one or more PDB structures (probes). Symmetry constraints (blue arrows; optional) can be enforced in a bash-shell script using the *pdbsymm* utility or any similar tool provided by the user. The resulting docked complex can be inspected in the external graphics program.

The new *collage* tool and external symmetry-manipulation programs can be combined to impose symmetry constraints on the fragments during multi-fragment docking. Fig. 5 ▶ provides an overview of the workflow. Standard volumetric map formats are converted to cubic lattices in *Situs* format with the *map*2*map* utility. Subsequently, the volume data are inspected and, if necessary, prepared for the fitting using a variety of map tools (§[Sec sec8]8). A data-type conversion (§[Sec sec3]3) is optional. The fitting of multiple PDB input files to the target map is handled by *collage* as described in §[Sec sec6]6. Symmetry constraints are outsourced to a separate program in the Unix bash-shell script. The *Situs*-native *pdbsymm* tool allows the generation of multiple symmetry-related copies following symmetry-axis conventions based on the target map. *C*, *D* and* H* (helical) symmetry options are currently supported. For other specialized cases (*e.g.* crystallographic symmetry), an alternative program (such as the *CCP*4 *pdbset* tool; http://www.ccp4.ac.uk/html/pdbset.html) may be substituted for *pdbsymm* in the workflow (Fig. 5 ▶). The entire Unix bash-shell implementation then proceeds as follows.

(i) Define which input structure is the initial master copy.
(ii) Generate symmetry mates from the master with *pdbsymm* (or alternative).
(iii) Extract individual symmetry mates from the output file using the Unix grep command (if necessary).
(iv) Refine all symmetry mates with *collage* using only a single conjugate-gradient step. Save master copy.
(v) Repeat (loop) steps (ii)–(iv) several times (check convergence).
(vi) Generate final symmetry mates from the master copy using *pdbsymm* (or alternative).

The goal of the scripting approach is to keep *Situs* tools modular and to avoid having to write specialized tools for every possible symmetry scenario. This means that *collag*e will technically still treat each fragment as independent. However, *collage* will take only a single conjugate-gradient step (step iv), after which the symmetry will again be enforced (in steps ii and vi). The net effect after several iterations of the loop is a symmetry-enforced conjugate-gradient optimization. An example of this approach is available online in the multi-fragment tutorial (file run_tutorial.bash at http://situs.biomachina.org/tutorial_multi.html).

**Figure 6:**
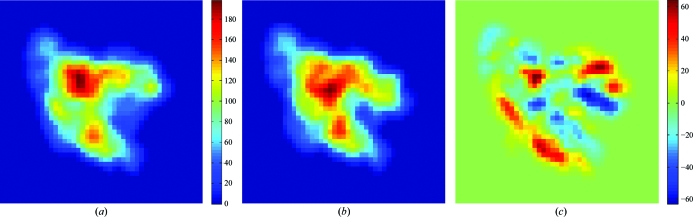
Two-dimensional projections and their difference. (*a*) Projection of a 15 Å resolution map of RNA polymerase in ‘open’ conformation (file 0_rnap2.situs of the flexible-fitting tutorial at http://situs.biomachina.org) computed with *voledit*. (*b*) Projection of the atomic structure of RNA polymerase in ‘closed’ conformation (file 0_rnap1.pdb), resolution lowered to 15 Å with *pdb*2*vol*. (*c*) Projection of the difference map created with *voledit* and *voldiff*. The images were rendered with *MATLAB* (http://www.mathworks.com/products/matlab).

A new function was added to the *voledit* tool in *Situs* v.2.6 that conveniently allows the user to render two-dimensional projections in addition to volume slices. Such projections are useful when comparing three-dimensional maps or resolution-lowered atomic structures with two-dimensional micrographs. Fig. 6 ▶ shows that discrepancies between two conformations can easily be detected in two dimensions. The projections (or slices) saved by *voledit* can be visualized by an external plotting program. *Situs* is mainly a three-dimensional package and does not have a tool for computing differences between two-dimensional images, but the difference of two two-dimensional projections is identical to the projection of the three-dimensional difference map, which can be computed with *voldiff*. The entire workflow used to create Fig. 6 ▶ consists of several steps that are most efficiently implemented in a shell script as follows.

(i) Create a low-resolution map from the atomic probe structure using *pdb*2*vol*.
(ii) Match the map size to that of the comparison map by cropping and zero padding using *voledit*.
(iii) Create the projection of both probe and comparison maps using *voledit*.
(iv) Create a difference map with *voldiff*.
(v) Create the projection of the difference map using *voledit*.

The shell script example (Fig. 6 ▶) is available online in part II of the flexible docking tutorial (http://situs.biomachina.org/tutorial_flex2.html).

## 8. Discussion

The *Situs* software consists of multiple standalone tools that can be combined in various creative ways. The modular design offers great advantages to the user who wishes to take advantage of shell-scripting capabilities. To encourage experimentation, bash scripts are included with all online tutorials. This overview also summarizes important conventions that form the basis of *Situs* functionality.

One active research area that exceeds the scope of this article is flexible docking, which bring deviating features of multi-resolution structures into register (Wriggers *et al.*, 2004 ▶; Wriggers, 2010 ▶). Fig. 6 ▶ shows the differences between flexed structures of RNA polymerase in the form of two-dimensional projections. Systematic tests and validations of flexible fitting with spatial interpolation have been carried out using the *qplasty* tool (Rusu *et al.*, 2008 ▶) and experimental applications of flexible docking (using molecular-dynamics refinement) were performed in collaboration with experimental laboratories on systems such as RNA polymerase (Darst *et al.*, 2002 ▶) and the thick filament of tarantula muscle (Alamo *et al.*, 2008 ▶).

Another noteworthy application that exceeds the scope of this paper is ‘volume algebra’, in which map densities are modified according to simple algebraic operations (Wriggers *et al.*, 2011 ▶). Volume-algebra operations are typically enabled by map-density registration (Fig. 1 ▶) and include map summation or averaging (*volaver*), difference mapping (*voldiff*), binary masking and map multiplication (*voledit*, *volmult*) and density matching using an affine transformation (*volhist*), as well as cropping, thresholding and segmentation (*voledit*). For application examples of volume algebra operations, see Fig. 1 in Wriggers *et al.* (2011 ▶) and the online tutorials.

The performance of multi-fragment-based refinement (Figs. 4 ▶ and 5 ▶) is the subject of ongoing research. Our empirical tests with experimental maps have shown that the refinement is very robust. The normalization of the cross-correlation coefficient (1) penalizes steric clashes between fragments, similar to features of a tabu search using genetic algorithms (Rusu & Birmanns, 2010 ▶). Also, the radius of convergence for the method appears to be rather large, reducing the need for exhaustive exploration. The performance will be evaluated further in future work.

*Situs* has been ported to multiple platforms and the source code is freely available at http://situs.biomachina.org.

A comparison of various *CCP*4-derived map formats and related *Situs* read and write conventions are provided in the Supplementary Material^1^.

## Supplementary Material

Supplementary material file. DOI: 10.1107/S0907444911049791/ba5170sup1.pdf

## Footnotes

1 Supplementary material has been deposited in the IUCr electronic archive (Reference: BA5170). Services for accessing this material are described at the back of the journal.
