# Supplementary material for: Conventions and workflows for using Situs
Source: Acta Crystallogr D Biol Crystallogr. 2012 Mar 16;68(Pt 4):344–51. doi: 10.1107/S0907444911049791 (PMC3322594; doi:10.1107/S0907444911049791)
Supplement: Supplementary file 1 [file d-68-00344-sup1.pdf]

Supplementary Data

“Conventions and work flows for using *Situs*” by Willy Wriggers

Acta Crystallographica Section D (2012)

(Proceedings of the CCP4 Study Weekend 2011)

Situs, CCP4 and MRC 3D Density Map File Formats and Format Conventions

Revision: 28-Feb-12

Content:

1. *Situs* Format ……………………………………………………..…. 2

2. *CCP4* Format …………………………………………………….. 3

3. *MRC* Format (pre-2000, obsolete) …………………………….... 4

4. *MRC 2000* Format ……………………………………….……..... 5

5. Other *MRC* Variants ……………………………...………......… 6

6. Format Conventions ….………………...…………………......... 7

7. Future Updates ……………………...…….……………….…..... 8

1. *Situs* Format (current)

Source: Author

The *Situs* map format was once necessary to keep track of the 3D coordinate system within the *Situs* package (see main article), to enforce a cubic lattice, and to be independent of the format variations in the community (6.). The advantages of the format are:

- It is unambiguous and requires only a minimalist header: In the map format, a short header holds the voxel spacing WIDTH, the map origin as defined by the 3D coordinates of the first voxel ORIGX, ORIGY, ORIGZ, and the map dimensions (number of increments) NX, NY, NZ. The header is followed by data fields such that X increments change fastest and Z increments change slowest.
- It is ASCII-based and thus easily readable/editable with a text editor.
- It is supported by the molecular graphics programs *VMD*, *Chimera*, and *Sculptor*, by the *EMAN2* reconstruction package, and also by the *em2em* format conversion tool.

Disadvantages of the *Situs* format (compared to typical binary formats) are:

- The file size is about 3 times larger than that of comparable binary formats, requiring compression of archived maps.
- Very large maps take noticeable time to read from (or write to) hard disk.
- A separate format conversion step was needed in the *Situs* work flow (*map2map* tool).

To take advantage of binary data storage, *CCP4* and *MRC* file formats were recently adopted for direct use within *Situs* programs. In recent years, *CCP4*-derived formats (such as *MRC*) added non-crystallographic capabilities to keep track of the 3D coordinate system, enabling their use within *Situs*. The original *CCP4* format was also adopted as the official format of the Electron Microscopy Data Bank (EMDB). Unfortunately, the various implementations of the *CCP4* / *MRC* format family (sections 2.-5.) are not consistent. The goal of *Situs* development was to automate many of the necessary transformations in a way that is transparent to the user (6.).

2. *CCP4* Format (current)

Source: http://www.ccp4.ac.uk/html/maplib.html

```
Map/Image Header
Length = 1024 bytes, organized as 56 words followed by space for 10 80 byte text labels.

 1      NC              # of Columns   (fastest changing in map)
 2      NR              # of Rows
 3      NS              # of Sections  (slowest changing in map)
 4      MODE            Data type
                          0 = envelope stored as signed bytes (from -128 lowest to 127 highest)
                          1 = Image    stored as Integer*2 (16-bit)
                          2 = Image    stored as Reals (32-bit)
                          3 = Transform       stored as Complex Integer*2
                          4 = Transform       stored as Complex Reals
                          5 == 0

                          Note: Mode 2 is the normal mode used in the CCP4 programs.
                                Other modes than 2 and 0 may NOT WORK.

 5      NCSTART         Number of first COLUMN  in map
 6      NRSTART         Number of first ROW     in map
 7      NSSTART         Number of first SECTION in map
 8      NX              Number of intervals along X
 9      NY              Number of intervals along Y
10      NZ              Number of intervals along Z
11      XLENGTH         Cell Dimensions (Angstroms)
12      YLENGTH                     "
13      ZLENGTH                     "
14      Alpha           Cell Angles (Degrees)
15      Beta                        "
16      Gamma                       "
17      MAPC            Which axis corresponds to Columns  (1,2,3 for X,Y,Z)
18      MAPR            Which axis corresponds to Rows     (1,2,3 for X,Y,Z)
19      MAPS            Which axis corresponds to Sections (1,2,3 for X,Y,Z)
20      AMIN            Minimum density value
21      AMAX            Maximum density value
22      AMEAN           Mean density value (average)
23      ISPG            Space group number
24      NSYMBT          Number of bytes used for storing symmetry operators
25      LSKFLG          Flag for skew transformation, =0 none, =1 if it follows
26-34   SKWMAT          Skew matrix S (in order S11, S12, S13, S21 etc) if
                        LSKFLG .ne. 0.
35-37   SKWTRN          Skew translation t if LSKFLG .ne. 0.
                        Skew transformation is from standard orthogonal
                        coordinate frame (as used for atoms) to orthogonal
                        map frame, as

                        Xo(map) = S * (Xo(atoms) - t)

38-52   future use      Storage space sometimes used by specific programs (default = 0).
53      MAP             Character string 'MAP ' to identify file type
54      MACHST          Machine stamp (indicating the machine type which wrote file)
55      ARMS            Rms deviation of map from mean density
56      NLABL           Number of labels being used
57-256  LABEL(20,10)    10 80-character text labels (ie. A4 format)


Symmetry records (see NSYMBT) follow - if any - stored as text as in International Tables,
operators separated by * and grouped into 'lines' of 80 characters (ie. symmetry operators do not
cross the ends of the 80-character 'lines' and the 'lines' do not terminate in a *).

Data records follow.
```

3. *MRC* Format (pre-2000, obsolete)

Source: *MRC* Cambridge Image Processing System (22-Mar-93)

Significant changes vs. *CCP4* shown in bold / blue

```
Map/Image Header
Length = 1024 bytes, organized as 56 words followed by space for 10 80 byte text labels.

 1      NX              # of Columns   (fastest changing in map)
 2      NY              # of Rows
 3      NZ              # of Sections  (slowest changing in map)
 4      MODE            Data type (typically 0 and 2 are used for 3D Maps, others may not work)
                          0 = Image    stored as Integer*1 (8-bit)
                              Note 1: Values between 127 & 255 stored as -128 to -1 but
                              returned within MRC software as their original (unsigned) values.
                              Note 2: Programs that support pre-2000 MRC typically follow this
                              “unsigned” convention.
                          1 = Image    stored as Integer*2 (16-bit)
                          2 = Image    stored as Reals (32-bit)
                          3 = Transform       stored as Complex Integer*2
                          4 = Transform       stored as Complex Reals

 5      NXSTART         Number of first COLUMN  in map (Default = 0)
 6      NYSTART         Number of first ROW     in map      "
 7      NZSTART         Number of first SECTION in map      "
 8      MX              Number of intervals along X
 9      MY              Number of intervals along Y
10      MZ              Number of intervals along Z
11      XLENGTH         Cell Dimensions (Angstroms)
12      YLENGTH                      "
13      ZLENGTH                      "
14      Alpha           Cell Angles (Degrees)
15      Beta                         "
16      Gamma                        "
17      MAPC            Which axis corresponds to Columns  (1,2,3 for X,Y,Z)
18      MAPR            Which axis corresponds to Rows     (1,2,3 for X,Y,Z)
19      MAPS            Which axis corresponds to Sections (1,2,3 for X,Y,Z)
20      AMIN            Minimum density value
21      AMAX            Maximum density value
22      AMEAN           Mean density value (average)
23      ISPG            Space group number (0 for images)
24      NSYMBT          Number of bytes used for storing symmetry operators (typically 0 or 80)
25-53   EXTRA           Extra, user defined storage space (default = 0).
54      XORIGIN         X origin (2D image origin, generally used for relating phase origins)
55      YORIGIN         Y origin (2D image origin, generally used for relating phase origins)
56      NLABL           Number of labels being used
57-256  LABEL(20,10)    10 80-character text labels (ie. A4 format)

Symmetry records (see NSYMBT) follow - if any - stored as text as in International Tables,
operators separated by * and grouped into 'lines' of 80 characters (ie. symmetry operators do not
cross the ends of the 80-character 'lines' and the 'lines' do not terminate in a *).

Data records follow.
```

4. *MRC 2000* Format (current)

Source: http://www2.mrc-lmb.cam.ac.uk/image2000.html

Significant changes vs. old *MRC* only shown in bold / blue

Significant changes vs. both *CCP4* and old *MRC* shown in bold / green

```
Map/Image Header
Length = 1024 bytes, organized as 56 words followed by space for 10 80 byte text labels.

 1      NX              # of Columns   (fastest changing in map)
 2      NY              # of Rows
 3      NZ              # of Sections  (slowest changing in map)
 4      MODE            Data type (typically 0 and 2 are used for 3D Maps, others may not work)
                          0 = Image signed 8-bit bytes, range -128 to 127
                              Note 1: Many software programs now support signed 8-bit bytes at
                              face value, similar to CCP4. Examples: EMAN2 and Chimera.
                              Note 2: MRC, however, still uses the pre-2000 “unsigned” convention
                              (see 3. above); info from Judith Short, MRC Cambridge, UK.
                          1 = Image    16-bit halfwords
                          2 = Image    32-bit reals
                          3 = Transform       complex 16-bit integers
                          4 = Transform       complex 32-bit reals

                        (Data types same as pre-2000 MRC which uses FORTRAN nomenclature)

 5      NXSTART         Number of first COLUMN  in map (Default = 0)
 6      NYSTART         Number of first ROW     in map      "
 7      NZSTART         Number of first SECTION in map      "
 8      MX              Number of intervals along X
 9      MY              Number of intervals along Y
10      MZ              Number of intervals along Z
11-13   CELLA           Cell Dimensions (Angstroms)
14-16   CELLB           Cell Angles (Degrees)
17      MAPC            Which axis corresponds to Columns  (1,2,3 for X,Y,Z)
18      MAPR            Which axis corresponds to Rows     (1,2,3 for X,Y,Z)
19      MAPS            Which axis corresponds to Sections (1,2,3 for X,Y,Z)
20      DMIN            Minimum density value
21      DMAX            Maximum density value
22      DMEAN           Mean density value (average)
23      ISPG            Space group number 0 or 1 (default = 0)
24      NSYMBT          Number of bytes used for storing symmetry operators (0 or 80)
25-49   EXTRA           Extra, user defined storage space (default = 0).
50      XORIGIN         X origin used for transforms
51      YORIGIN         Y origin used for transforms
52      ZORIGIN         Z origin used for transforms
53      MAP             Character string 'MAP ' to identify file type
54      MACHST          Machine stamp (indicating the machine type which wrote file)
55      RMS             Rms deviation of map from mean density
56      NLABL           Number of labels being used
57-256  LABEL(20,10)    10 80-character text labels

Symmetry records (see NSYMBT) follow - if any - stored as text as in International Tables,
operators separated by * and grouped into 'lines' of 80 characters (ie. symmetry operators do not
cross the ends of the 80-character 'lines' and the 'lines' do not terminate in a *).

Data records follow.
```

5. Other *MRC* Variants

A. Minor variations of the obsolete, pre-2000 *MRC* format, are known:

- the start values of the map were not set correctly to properly reflect the origin
- the cell axes were given in voxel units, not in Angstrom
- confusingly, some pre-2000 *MRC* maps also had the *CCP4* style 'MAP' string set

Such maps are rarely encountered today.

B. Some software packages use their own variation of the above formats. For example, the *IMOD* package used in tomography supports various non-standard data modes and additional header fields relevant to tomography. The cell axes may be given in nm units, not in the traditional Angstrom units. Also, *IMOD* files currently follow the "unsigned" 8-bit byte convention (see MODE 0 in 3.), but due to compatibility problems with the EMDB (which uses *CCP4*), the format may soon adopt signed 8-bit bytes. For more details on the *MRC* format variants used by *IMOD* see http://bio3d.colorado.edu/imod/doc/mrc_format.txt.

Although the conventions described in the following section were designed specifically for *MRC 2000* and *CCP4* formats, they are tolerant of variations such as *IMOD* and pre-2000 *MRC*. It should be noted that non-standard header values (e.g. due to different unit conventions) can be edited manually with the *map2map* program, if necessary.

6. Format Conventions

The developers of *em2em* (Michael Schatz), *Chimera* (Tom Goddard), *VMD* (John Stone), *Sculptor* (Stefan Birmanns), and *Situs* (Willy Wriggers) discussed various implementation details of the *CCP4* and *MRC 2000* formats in the spring of 2009 to coordinate a consistent map representation across different software. Incremental changes and the lack of utility of some *CCP4* features to the EM community have caused the pre-2000 *MRC* format to become incompatible with *CCP4* (see 3.), but the more recent *MRC 2000* format (4.) reintroduced some *CCP4* header fields and conventions (in addition to new ones) so that it might be possible to merge the two formats in practical applications. There is good reason to do this: Whether a file is *CCP4* or *MRC 2000* may get lost in vague file suffixes (.dat, .map, etc.). Also, sometimes X-ray maps are viewed or written by EM tools, and vice-versa.

The developers discussed four remaining differences between the formats and thought of ways to handle them gracefully: the origin conventions, the axis permutation capability, the support for non-cubic lattices, and the support of signed or unsigned 8-bit bytes. The following conventions were adopted by *Situs* on automatic read and write of *CCP4* and *MRC 2000* maps.

A. Origin Conventions

CCP4 maps are in register with the coordinate system origin, and only N*START indices are set, whereas the *MRC 2000* *ORIGIN (fields 50-52) are typically set to zero. In contrast, *MRC 2000* uses *ORIGIN for identifying the position of the first voxel, and the map is not necessarily in register with the origin of the coordinate system.

- **Read -** auto detect *MRC 2000* or *CCP4* style origin:

If *ORIGIN = (0,0,0), use N*START, i.e. define the coordinate of the first voxel as N*START · *LENGTH / M* (also takes care of cases where both N*START and *ORIGIN are zero)

If *ORIGIN != (0,0,0), define the coordinate of the first voxel as *ORIGIN (also takes care of cases where both N*START and *ORIGIN are non-zero)

- **Write -** maximize compatibility of the output map:

If the map is in register with the origin of the coordinate system, i.e. the origin is an integer multiple of the grid spacing (RESOL* = *LENGTH / M*), support both conventions:

set *ORIGIN = *ORIGIN
set N*START = *ORIGIN · M* / *LENGTH

else support only *MRC 2000* (and give warning):

set *ORIGIN = *ORIGIN
set N*START = 0

Notes: Fields above correspond to *MRC 2000* nomenclature; * is a wildcard for X, Y or Z.

B. Axis Permutation

It depends mainly on the user community whether axis permutations are supported via the MAP[C,R,S] fields or not. The crystallographic community (*CCP4*) definitely supports it, but the EM community (*MRC*) tends to ignore these fields and assumes MAP[C,R,S]=1,2,3. *Situs* generally supports axis permutations. Due to the possible mix of software there is good reason to support *CCP4* style axis mapping even to files believed to be in *MRC* format.

Note, that when permuting X,Y,Z axes (order of data records) via MAP[C,R,S], one must permute also the fields N[C,R,S] and N[C,R,S]START in the header. The *CCP4* header clearly separates the permutable C,R,S fields from the fixed X,Y,Z fields. In *MRC 2000* one must then permute the equivalent N[X,Y,Z] and N[X,Y,Z]START fields, even though these use X,Y,Z designation.

C. Non-Cubic Lattices

There are additional complexities in the case of skewed unit cells and non-cubic lattices. As with axis permutation, skewed unit cells are generally supported, even in *MRC 2000* maps. Skew parameters and voxel spacing parameters are computed from the cell angles and dimensions (fields 8-16), ignoring the *CCP4* fields 25-37.

D. Signed or Unsigned 8-Bit Bytes

Input maps in MODE 0 use 8-bit bytes for space-saving data storage. The *CCP4* standard supports signed values in the range of -128 to 127. However, the *MRC* programs internally convert negative values (-128 to -1) to positive values (127 to 255) by adding 256. This “unsigned” convention was once universally adopted for *MRC* maps prior to 2000 (see 3.) but many programs in the EM community now interpret signed 8-bit bytes at face value (see 4.). Therefore, *Situs* determines the convention on automatic read of MODE 0 maps, based on a simple discriminant analysis of the density values. Output maps are automatically written in MODE 2, except if MODE 0 is explicitly requested during manual use of the *map2map* program.

Note that *Situs* automatically recognizes the endianism of MODE 1 or MODE 2 maps using the numeric values of the header fields. If a MACHST machine stamp (field 54) is present in imported files, it is ignored. For compatibility reasons, the correct *CCP4* / *MRC 2000* machine stamp is set in exported maps.

The above *Situs* conventions are (mostly) supported by *Sculptor*, *VMD*, *Chimera*, and *em2em*. The area where *em2em* currently does not comply is in assuming cubic lattices and in not allowing axis permutation, owing to the EM focus of the software. *VMD* does not presently support MODE formats other than 2. *Chimera* distinguishes more strictly than *Situs* between certain *CCP4*/*MRC* flavors. *Situs* is unique in detecting the 8-bit signed-ness convention. For more information see the respective user guides.

7. Future Updates

This document summarizes the map format conventions of *Situs* at the time of this writing (version 2.7). As the *MRC*-related formats continue to evolve, it is possible that future versions of the software require an update of this document, which will be posted online at http://situs.biomachina.org/fmap.pdf.
